# An Exploratory Study on Injury Patterns and Clinical Characteristics of Sports-Related Oral and Maxillofacial Trauma: A Single-Center Retrospective Study

**DOI:** 10.3390/cmtr19020029

**Published:** 2026-06-22

**Authors:** Kentaro Ayasaka, Yuhei Matsuda, Hiroto Tatsumi, Masako Fujioka-Kobayashi, Shota Norioka, Reon Morioka, Michitaka Somoto, Rie Sonoyama-Osako, Takahiro Kanno

**Affiliations:** 1 Department of Oral and Maxillofacial Surgery, Shimane University Faculty of Medicine, 89-1, Enya-Cho, Izumo 693-8501, Shimane, Japan; ayasakakentaro@med.shimane-u.ac.jp (K.A.); yuhei@med.shimane-u.ac.jp (Y.M.); tatsumi@med.shimane-u.ac.jp (H.T.); masako@med.shimane-u.ac.jp (M.F.-K.); shota.n-kdu@med.shimane-u.ac.jp (S.N.); moriokareon@med.shimane-u.ac.jp (R.M.); somoto.m@med.shimane-u.ac.jp (M.S.); r.osako@med.shimane-u.ac.jp (R.S.-O.); 2 Department of Medical Oncology, Shimane University Hospital, 89-1, Enya-Cho, Izumo 693-8501, Shimane, Japan; 3 Department of Pharmacology, Shimane University Faculty of Medicine, 89-1, Enya-Cho, Izumo 693-8501, Shimane, Japan

**Keywords:** oral and maxillofacial trauma, sports-related injury, orbital wall fracture, nasal bone fracture, naso-orbito-ethmoidal fractures, energy density

## Abstract

Sports-related oral and maxillofacial trauma exhibits distinct injury mechanisms depending on the type of sport; however, comprehensive analyses integrating these characteristics remain limited. This study aimed to elucidate the clinical features of sports-related oral and maxillofacial trauma. This single-center retrospective study included 1615 patients (mean age 41.04 years; 64.8% male) treated between 2012 and 2025 in Shimane University Hospital, Department of Oral and Maxillofacial Surgery/Maxillofacial Trauma Center, Shimane, Japan. Among them, 239 (14.8%) had sports-related injuries and were significantly younger and more frequently male (*p* < 0.001). Multivariable analysis showed that nasal bone (odds ratio [OR] 5.900, 95% confidence interval [CI] 3.309–10.521), orbital wall (OR: 8.044, 95% CI: 4.664–13.874), zygomatic and zygomatic arch (OR: 3.239, 95% CI: 1.455–7.213), and naso-orbito-ethmoidal fractures (OR: 6.507, 95% CI: 1.971–21.483), as well as contusions (OR: 2.601, 95% CI: 1.385–4.885), were positively associated, whereas mucosal lacerations (OR: 0.485, 95% CI: 0.290–0.811) and referral from dental/medical clinics (OR: 0.312, 95% CI: 0.178–0.546) were negatively associated (all *p* < 0.01). However, panfacial fractures occurred less frequently. Sports-related trauma was primarily associated with nasal, orbital wall, zygoma and zygomatic arch, and naso-orbito-ethmoidal fractures. These findings indicate that sports-related oral and maxillofacial trauma is characterized by distinct and predominantly midfacial fracture patterns.

## 1. Introduction

Oral and maxillofacial trauma (OMFT) accounts for approximately 18.5% of all traumatic injuries and is considered a common form of systemic trauma [1]. Such trauma arises from a variety of etiologies, including traffic accidents, falls, interpersonal violence, and sports-related activities, and encompasses a wide spectrum of injury patterns, including maxillofacial fractures, dental injuries, and intraoral and extraoral soft tissue injuries [2,3]. Maxillofacial trauma not only results in the impairment of stomatognathic functions, such as visual disturbance, occlusion, mastication, swallowing, and articulation, but also leads to esthetic concerns, including facial deformity, thereby adversely affecting patients’ quality of life (QoL) [4]. Ukpong et al. reported that QoL significantly deteriorates following maxillofacial trauma, with a high incidence of depressive symptoms observed during the post-trauma follow-up period. Sequelae in patients with maxillofacial fractures have been reported to include facial nerve paralysis, diplopia, blindness, malocclusion, trismus, globe malposition, malar flattening, facial contour changes, and wound infections [4]. Therefore, a comprehensive understanding of injury patterns in maxillofacial trauma, along with appropriate management, is essential not only for the restoration of stomatognathic function but also for facilitating patients’ social reintegration and recovery of daily life.

In recent years, with widespread participation in sports and an increasing number of athletes, the incidence of sports-related injuries has increased [5]. Sports-related OMFT occurs through mechanisms specific to each sport, including physical contact between players, collisions with equipment, and falls. Compared with trauma caused by traffic accidents or interpersonal violence, sports-related injuries are highly dependent on the characteristics of each sport in terms of injury circumstances and mode of external force application. Previous studies have reported that sports-related OMFT predominantly affects young males and is characterized by distinct fracture patterns, particularly involving the midface [6]. Consequently, the causative sports vary considerably across countries and regions. Bojino et al. conducted a single-center retrospective study of 3231 cases of maxillofacial fractures in Italy, a European country, and reported that 432 cases were attributable to sports-related trauma. The most common causative sport was football/soccer (234 cases, 54.2%), followed by skiing (54 cases, 12.5%) and equestrian sports (33 cases, 7.6%) [7]. In contrast, in a single-center retrospective study of 1253 cases of maxillofacial fractures in the United States, Audlin et al. reported that 33 cases of sports-related trauma occurred among individuals aged 13–19 years, with baseball accounting for 10 cases (30.3%) and basketball for 7 cases (21.2%) [8]. Furthermore, in Switzerland, where winter sports are highly popular, Exadaktylos et al. conducted a single-center retrospective study of 90 cases of sports-related maxillofacial trauma and reported that skiing was the most common cause (23 cases, 25.6%), followed by cycling (19 cases, 21.1%), and football/soccer (12 cases, 13.3%) [9]. Taken together, these findings suggest that the sports that cause OMFT exhibit regional and/or country specificity, reflecting local sporting culture and participation patterns.

In our previous single-center retrospective study conducted in Japan, an East Asian country, involving 1203 patients with maxillofacial trauma, the most common cause of injury was slip down (593 cases, 49.3%), followed by traffic-related trauma (188 cases, 15.6%) and sports-related trauma (178 cases, 14.8%), indicating that sports represent the third most frequent mechanism of injury in this population of Japan [10]. Additionally, participation in physical activity is relatively high in Japan. According to a 2020 survey conducted by the Agency for Cultural Affairs, 67.1% of junior high and high school students participate in extracurricular sports club activities after school [11]. Regarding sports-related maxillofacial fractures in Japan, Iida et al. reported a single-center retrospective study of 146 cases in which baseball was the most common causative sport (54 cases, 37.0%), followed by rugby (26 cases, 17.8%) and football/soccer (15 cases, 10.3%). Mandibular fractures were the most frequent (92 patients, 36.1%), followed by midfacial fractures (37 patients, 25.3%) [12]. Similarly, Sawaki et al. reported a single-center retrospective study of 60 cases of sports-related maxillofacial fractures in Japan, of which baseball was the most common cause (20 cases, 33.3%), followed by rugby (seven cases, 11.7%). In terms of fracture patterns, mandibular fractures accounted for the majority (41 cases, 68.3%), whereas maxillary fractures were relatively infrequent (5 cases, 9.3%) [13]. Therefore, even within Japan, interregional and interinstitutional variability exists in the epidemiology of sports-related maxillofacial trauma, making it challenging to accurately characterize the overall injury patterns. A literature review suggests that common features of sports-related maxillofacial fractures in Japan include baseball and rugby as major causative sports and a high proportion of mandibular fractures [14].

However, interpretation of these findings requires consideration of the healthcare delivery system for OMFT treatment and care in Japan. Management of maxillofacial fractures is undertaken not only by oral and maxillofacial surgeons but also by plastic surgeons and otorhinolaryngologists. Consequently, studies limited to a single specialty may reflect a case-selection bias based on the scope of practice in that discipline. Specifically, studies conducted primarily in oral and maxillofacial surgery departments tend to include a higher proportion of maxillary and mandibular fractures, whereas those conducted in plastic surgery or otorhinolaryngology departments are more likely to report fractures of the nasal bone, orbital wall, zygoma and zygomatic arch, and other midfacial structures [15]. Although this division of clinical responsibilities is a rational feature of the Japanese healthcare system, it poses a challenge for comprehensive epidemiological assessment. As a result, studies that systematically evaluate all types of maxillofacial fractures—including maxillary, mandibular, nasal, orbital wall, zygoma and zygomatic arch, and other midfacial fractures—within a unified analytical framework remain limited to date [16].

At the Maxillofacial Trauma Center of Shimane University Hospital, a tertiary referral center in the region, a clinical system has been established in which most patients with maxillofacial trauma are referred and managed. Therefore, although the findings may have been influenced by regional characteristics, this setting provides a relatively reliable framework for observing the epidemiology and injury patterns of OMFT. Based on the hypothesis that injury patterns and anatomical predilection sites differ according to the type of sport, this study focused on sports-related cases in patients with OMFT. Accordingly, this study aimed to elucidate the clinical characteristics of sports-related OMFT.

## 2. Patients and Methods

This study was approved by the Medical Research Ethics Committee of the Shimane University Faculty of Medicine (approval no.: 20151201–1). All the procedures were performed in accordance with the principles of the Declaration of Helsinki.

### 2.1. Data Collection

This study enrolled patients who presented to the Department of Oral and Maxillofacial Surgery and the Maxillofacial Trauma Center at Shimane University Hospital between 1 April 2012, and 31 December 2025. Patients diagnosed with OMFT and treated by the same single surgical team under a consistent treatment policy were included in the study, whereas those with incomplete data were excluded. This was a retrospective observational study, and clinical data were extracted from electronic medical records.

#### 2.1.1. Background Data

The following variables were obtained from the electronic medical records: age (years) and sex (male/female).

#### 2.1.2. Types of Oral and Maxillofacial Trauma

Trauma-related variables were classified as follows: injury status (contusion; laceration [mucosa/skin], tooth injury, alveolus fracture, maxillofacial fracture [maxilla, mandibular body, mandibular condyle, mandibular body and mandibular condyle, nasal bone, naso-orbito-ethmoidal fracture (NOE), orbital wall, zygoma and zygomatic arch, and panfacial maxillofacial fractures]). Types of OMFT were classified according to the AO Surgery Reference [17].

#### 2.1.3. Referral Origin

The sources of referrals were dental/medical clinics, direct visits, emergency and critical care center (ECC) and advanced trauma center (ATC), * *p* < 0.05, and other departments.

#### 2.1.4. Mode of Transportation to the Hospital

The modes of transportation were classified as ambulance, doctor’s helicopter, and on foot.

#### 2.1.5. Cause of Injury

The causes of injury were categorized as falls, slip downs, sports, traffic accidents, violence, work-related accidents, and others.

#### 2.1.6. Sports Accidents

Sports were categorized into four disciplines—namely, the top three sports (baseball, basketball, football/soccer) identified in materials from the Japan High School Athletic Federation, plus rugby (included due to its ball handling and physical contact)—and an “others” category [18].

### 2.2. Statistical Analysis

For patient background characteristics, continuous variables were assessed for normality using the Shapiro–Wilk test. Descriptive statistics are presented as mean (standard deviation) for continuous variables and as number of cases (%) for categorical variables. Group comparisons were conducted between the sports and non-sports groups using the *t*-test or chi-square test, as appropriate, for the type of variable. As the primary analysis, a multivariate logistic regression analysis (forced entry method) was performed with sports-related trauma as the dependent variable, adjusting for age and sex as potential confounders. As a sub-analysis, group comparisons among the major sports categories (baseball, basketball, football/soccer, rugby and others) were performed using the *t*-test or chi-square test, as appropriate, for the variable type. Subsequently, multivariate logistic regression analyses were conducted with participation in each sport as the dependent variable. All the statistical analyses were performed using SPSS version 29 (IBM Corp., Armonk, NY, USA). Two-tailed *p*-values were calculated for all analyses with the alpha level of significance set at 0.05.

## 3. Results

### 3.1. Descriptive Statistics of Patients and Group Comparisons of the Non-Sports and Sports Groups

Among the 1615 patients, 239 (14.8%) had sports-related OMFT. Sports-related injuries were the third most common cause of injury. These patients were significantly younger and more likely to be male (*p* < 0.001). Sports-related trauma was characterized by a higher prevalence of nasal and orbital wall fractures, fewer mandibular condyle and panfacial fractures, and a greater tendency to present on foot than by ambulance (*p* < 0.01, Table 1).

### 3.2. Multivariable Analysis of Factors Associated with Sports-Related Trauma

In the multivariable analysis, younger age and male sex were independently associated with sports-related trauma (*p* < 0.001). Nasal bone and orbital wall fractures, as well as contusions, were positively associated, whereas mucosal lacerations and referral from dental/medical clinics were negatively associated (Table 2).

### 3.3. Sub-Analysis of Group Comparisons Across Major Sports Categories

The proportion of male patients differed significantly. Orbital wall fractures were more frequent in the baseball group, and tooth injuries were more common in the basketball group. In soccer, nasal bone fractures were the most common, whereas in rugby, orbital wall fractures were the most frequent (*p* < 0.05, Table 3).

### 3.4. Sub-Analysis of Multivariable Analysis Across Major Sports Categories

In the sub-analysis, maxillary fractures, mandibular body fractures, orbital wall fractures, and NOE fractures were positively associated with specific sports categories, whereas mucosal and skin lacerations and tooth injuries were negatively associated (*p* < 0.05, Table 4). Similar analyses were performed for basketball, football/soccer and rugby; however, no significant associations were identified.

## 4. Discussion

The characteristics of this study population and healthcare-seeking behavior indicated that sports-related OMFT were more frequently observed in young males. This finding is consistent with the background in Japan, where participation in sports activities is more common among young males. According to reports from the All Japan High School Athletic Federation (Koutairen), the most popular sports among male high school students in Japan are football/soccer, baseball, and basketball, in that order [18]. This distribution generally corresponded with the types of sports most frequently associated with injuries in the present study. Regarding the mode of hospital visit, sports-related OMFT were significantly less likely to involve referral origin from dental clinics and significantly more likely to present on foot. This suggests that even in cases where relatively severe injuries such as maxillofacial fractures were suspected, patients or their coaches or family members may have judged that ambulance transport was not necessary, while still recognizing the need for further evaluation and treatment at a tertiary care center. Consequently, patients may have directly visited a tertiary hospital accompanied by coaches or family members. In contrast, previous reports have indicated that approximately 73% of patients with systemically multiple trauma due to traffic accidents were transported by ambulance [19]. Therefore, presentation on foot rather than using emergency medical services may represent one of the characteristic features of sports-related OMFT. On the other hand, certain sports-related OMFT, maxillofacial fracture such as trapdoor-type orbital fractures with extraocular muscle entrapment, require urgent surgical intervention [20]. Accordingly, it is important to consider the potential severity of OMFT sustained during sports activities. Oral and maxillofacial surgeons should therefore raise awareness among coaches, family and relevant personnel regarding these risks and emphasize the importance of requesting emergency medical transport when necessary.

This study comprehensively analyzed the injury patterns and fracture sites in sports-related OMFT. Fractures of the nasal bone, orbital walls, zygomatic and zygomatic arches, and NOE were the predominant fracture types in sports-related injuries and occurred significantly more frequently than in non-sports-related trauma [6]. In contrast, mucosal and skin lacerations were less common, suggesting that sports-related trauma is characterized by localized impacts on the anterior midface rather than high-energy injuries involving the entire facial skeleton. These findings indicate the presence of distinct maxillofacial fracture patterns specific to sports-related trauma and suggest that differences in injury mechanisms substantially influence the clinical presentation [21]. Importantly, these associations were not observed across all sport-related OMFT. The positive associations were primarily limited to fractures involving the nasal bone, orbital wall, NOE region, and zygomatic/zygomatic arch, whereas panfacial fractures and mandible condylar fractures did not demonstrate independent associations after adjustment for potential confounders. In addition, although zygomatic and zygomatic arch fractures were more frequent in the non-sports group in the crude comparison, the association became positive after multivariable adjustment, suggesting that differences in patient characteristics, particularly age and sex, may have confounded the unadjusted findings.

The nasal bone and orbit are anatomically prominent structures in the anterior face, characterized by a thin cortical bone and limited cushioning from the overlying soft tissues, making them vulnerable to the direct transmission of external forces [2]. In particular, the orbital floor and medial wall consist of extremely thin bony structures and are prone to disruption when a sudden increase in intraorbital pressure occurs due to blunt trauma, resulting in so-called blowout fractures [22]. During sports activities, localized and instantaneous impact forces such as those from balls or player-to-player contact are often concentrated at a specific point on the face, and these anatomical characteristics likely contribute to the predilection for maxillofacial fractures in these regions [6]. Previous studies have also demonstrated that sports-related maxillofacial trauma predominantly affects young males and is characterized by midfacial fractures, which is consistent with the present findings and global epidemiological trends [6]. Furthermore, while traffic accidents and falls from heights are more likely to result in panfacial fractures, sports-related injuries tend to involve isolated and localized fractures, reflecting differences in external forces and mechanisms of energy transfer [21].

In the analysis with baseball-related injury, injuries related to baseball showed a significantly higher incidence of maxillary, mandibular body, orbital wall, and NOE fractures, highlighting a sports-specific risk profile. Hard balls have substantial mass and can reach high velocities during pitching or batting, resulting in considerable kinetic energy at impact; thus, direct facial blows may be more likely to occur, contributing to the increased incidence of these fractures [23]. This study further demonstrated that these findings likely reflect the characteristic mechanisms of external force application and energy transfer specific to sports-related trauma [6]. Understanding the mechanisms of injury in ball sports requires the consideration of both kinetic energy and energy density. The kinetic energy is expressed as E = 1/2 mv^2^ and is proportional to the square of the velocity. Thus, high-velocity projectiles possess substantial energy. Furthermore, energy density (energy per unit area) could be a critical determinant of injury severity because smaller contact areas result in greater localized energy concentrations [24]. For example, assuming a ball velocity of 100 km/h, a baseball (145 g) has approximately 56.0 J of kinetic energy, whereas a football/soccer ball (430 g) and a basketball (620 g) have approximately 166.0 J and 239.3 J, respectively. However, the contact areas differ markedly, at approximately 10 cm^2^ for baseball, 60 cm^2^ for football/soccer balls, and 80 cm^2^ for basketball. Consequently, the energy per unit area is substantially higher during baseball impacts, resulting in a localized concentration of force in the facial region. Indeed, calculated energy density values were 5.60 for baseball, 2.77 for basketball, and 2.99 for football/soccer; the calculated energy density values indicated that baseball generated the highest localized energy concentration. Therefore, the small size, high hardness, and high velocity of a ball for baseball may contribute to the occurrence of localized fractures. On the other hand, rugby was excluded from the above discussion because its ball is not a perfect sphere; however, as it involves more frequent physical contact than baseball, basketball, and football/soccer, different injury patterns were observed. These findings suggest that equipment and rules may also influence injury mechanisms.

The external force required to cause fractures of the oral and maxillofacial bones has been reported to differ by anatomical site, with approximately 30 G for nasal bone fractures, 50 G for zygomatic and zygomatic arch fractures, 100 G for maxillary fractures, 70–100 G for mandibular fractures, and 200 G for frontal bone fractures [25]. These findings indicate that the structural strength of oral and maxillofacial bones varies considerably depending on the location. In particular, the nasal bone and the orbital wall are relatively thin and fragile, making them more susceptible to fracture even under comparatively low external forces. In contrast, stronger bones such as the maxilla and frontal bone require greater forces to fracture. Considering these differences in bone strength, the localized impact generated by a baseball is sufficient to cause fractures of the nasal bone and periorbital structures, whereas it may not always be sufficient to fracture more robust bones such as the maxilla and frontal bone. Therefore, the findings of the present study are consistent with these theoretical thresholds.

Moreover, the predominance of localized fractures and the relative rarity of panfacial fractures in sports-related trauma contrasts with high-energy injuries such as traffic accidents and falls. This suggests that in sports-related trauma, the mode of energy concentration, rather than total energy, plays a critical role in determining injury patterns [21]. Accordingly, baseball can be considered a high-risk sport for maxillofacial trauma, and the present study provides an important clinical epidemiological quantification of this risk [23]. These findings are highly relevant from a preventive perspective. The use of face guards and protective masks, implementation of safety education, and modification of game rules have been suggested as effective primary prevention strategies to reduce the incidence of such injuries [3]. In particular, in school sports involving younger individuals, the introduction of protective equipment and reinforcement of supervision systems may help prevent long-term functional and esthetic impairments and reduce the associated healthcare burden [3].

Although this study has three limitations, including missing data owing to its single-center retrospective design, it has several strengths. First, these include the accumulation of a large number of cases over an extended period and direct comparison between sports-related and non-sports-related trauma with quantitative assessment of site-specific risks using multivariate analysis [21]. However, differences in interpersonal mechanisms of injury within the same sport were not considered, and further detailed analyses are warranted in future studies. Second, some injury categories, particularly NOE fractures, and sport-specific subgroups included a limited number of cases, which may have affected the stability of the regression estimates; therefore, these findings should be interpreted with caution and validated in larger multicenter studies. Third, some significant findings may represent false-positive findings due to multiple comparisons and should be interpreted cautiously. Oral and maxillofacial surgeons and other medical professionals treating oral and maxillofacial injuries caused by sports should conduct awareness campaigns on prevention tailored to the specific characteristics of each sport.

## 5. Conclusions

Compared to other etiologies, sports-related OMFT is associated with the occurrence of maxillofacial fractures. In baseball, the concentration of external force from a hard ball onto a localized area of the face may predispose vulnerable orbital wall bones to fractures. These findings suggest the need to promote preventive measures, including the use of sports-specific maxillofacial protective equipment.

## Figures and Tables

**Table 1:** Descriptive statistics of patients and between-group comparisons of the non-sports and sports groups (*N* = 1615).

			*N* (%) or Mean (SD)			
Item	Category	Sub-Category	Whole Data (*N* = 1615)	Non-Sports Group (*N* = 1376)	Sports Group (*N* = 239)	*p*-Value
Age (years)			41.04 (30.87)	45.07 (31.52)	17.79 (9.31)	<0.001 *
Sex	Male		1047 (64.8)	848 (61.6)	199 (83.3)	<0.001 *
	Female		568 (35.2)	528 (38.4)	40 (16.7)	
Fracture	Zygomatic and zygomatic arch		178 (11.0)	166 (12.1)	12 (5.0)	<0.001 *
	Orbital wall (s)		155 (9.6)	103 (7.5)	52 (21.8)	<0.001 *
	Nasal bone		143 (8.9)	105 (7.6)	38 (15.9)	<0.001 *
	Mandible	Mandibular body	30 (1.9)	23 (1.7)	7 (2.9)	0.192
		Mandibular condyle	53 (3.3)	51 (3.7)	2 (0.8)	0.017 *
		Mandibular body and mandibular condyle	49 (3.0)	47 (3.4)	2 (0.8)	0.038 *
	Panfacial		78 (4.8)	76 (5.5)	2 (0.8)	<0.001 *
	Maxilla		50 (3.1)	38 (2.8)	12 (5.0)	0.069
	NOE		19 (1.2)	12 (0.9)	7 (2.9)	0.015 *
Non-fracture	Laceration	Mucosa	265 (16.4)	240 (17.4)	25 (10.5)	0.006 *
		Skin	147 (9.1)	141 (10.2)	6 (2.5)	<0.001 *
	Tooth injury		286 (17.7)	247 (18.0)	39 (16.3)	0.583
	Contusion		86 (5.3)	65 (4.7)	21 (8.8)	0.018 *
	Alveolar fracture		76 (4.7)	62 (4.5)	14 (5.9)	0.406
Cause of injury	Slip downs		752 (46.6)	752 (54.7)	−	−
	Traffic accidents		240 (14.9)	240 (17.4)	−	−
	Sports	Baseball	106 (6.6)	−	106 (44.4)	−
		Basketball	35 (2.2)	−	35 (14.6)	−
		Football/soccer	24 (1.5)	−	24 (10.0)	−
		Rugby	18 (1.1)		18 (7.5)	
		Other sports	56 (3.5)	−	56 (23.4)	−
	Falls		221 (13.7)	221 (16.1)	−	−
	Violence		46 (2.8)	46 (3.3)	−	−
	Work related accidents		41 (2.5)	41 (3.0)	−	−
	Others		76 (4.7)	76 (5.5)	−	−
Referral origin	ECC/ATC		1190 (73.7)	1003 (72.9)	187 (78.2)	0.094
	Dental/medical clinic		209 (12.9)	192 (14.0)	17 (7.1)	0.003 *
	Other departments		135 (8.4)	112 (8.1)	23 (9.6)	0.448
	Direct visit		81 (5.0)	69 (5.0)	12 (5.0)	1.000
Mode of hospital visit	On foot		1092 (67.6)	875 (63.6)	217 (90.8)	<0.001 *
	Ambulance		490 (30.3)	469 (34.1)	21 (8.8)	<0.001 *
	Doctor helicopter		33 (2.0)	32 (2.3)	1 (0.4)	0.077

SD, standard deviation; NOE, naso-orbito-ethmoidal; ECC; emergency and critical care center, ATC; advanced trauma center, * *p* < 0.055.

**Table 2:** Multivariate logistic regression analysis with sports-related injury as the dependent variable.

			Univariate Analysis		Multivariate Analysis	
Item	Category	Sub-Category	OR (95%CI)	*p*-Value	OR (95%CI)	*p*-Value
Age (years)			0.963 (0.956–0.969)	<0.001 *	0.950 (0.941–0.959)	<0.001 *
Sex	Male		3.096 (2.169–4.425)	<0.001 *	2.591 (1.733–3.876)	<0.001 *
Fracture	Zygomatic and zygomatic arch		0.385 (0.211–0.704)	0.002 *	3.239 (1.455–7.213)	0.004 *
	Orbital wall (s)		3.437 (2.381–4.961)	<0.001 *	8.044 (4.664–13.874)	<0.001 *
	Nasal bone		2.288 (1.534–3.413)	<0.001 *	5.900 (3.309–10.521)	<0.001 *
	Mandible	Mandibular body	1.775 (0.753–4.184)	0.190		
		Mandibular condyle	0.219 (0.053–0.907)	0.036 *	0.878 (0.187–4.135)	0.870
		Mandibular body and mandibular condyle	0.239 (0.058–0.989)	0.048 *	1.725 (0.360–8.272)	0.495
	Panfacial		0.144 (0.035–0.592)	0.007 *	1.621 (0.342–7.684)	0.543
	Maxilla		1.861 (0.958–3.616)	0.067		
	NOE		3.430 (1.336–8.802)	0.010 *	6.507 (1.971–21.483)	0.002 *
Non-fracture	Laceration	Mucosa	0.553 (0.357–0.856)	0.008 *	0.485 (0.290–0.811)	0.006 *
		Skin	0.226 (0.098–0.517)	<0.001 *	0.426 (0.172–1.054)	0.065
	Tooth injury		0.891 (0.616–1.290)	0.542		
	Contusion		1.943 (1.164–3.243)	0.011 *	2.601 (1.385–4.885)	0.003 *
	Alveolar fracture		1.319 (0.726–2.396)	0.364		
Referral origin	ECC/ATC		1.337 (0.962–1.859)	0.084		
	Dental/medical clinic		0.472 (0.282–0.791)	0.004 *	0.312 (0.178–0.546)	<0.001 *
	Other departments		1.202 (0.750–1.925)	0.445		
	Direct visit		1.001 (0.534–1.878)	0.997		
Mode of hospital visit	On foot		5.648 (3.594–8.876)	<0.001 *	11.889 (1.403–100.743)	0.023 *
	Ambulance		0.186 (0.117–0.296)	<0.001 *	2.470 (0.283–21.581)	0.414
	Doctor helicopter		0.176 (0.024–1.298)	0.088		

NOE; naso-orbito-ethmoidal, ECC; emergency and critical care center, ATC; advanced trauma center, OR; odds ratio, CI; confidence interval, * *p* < 0.05.

**Table 3:** Sub-analysis of between-group comparisons by sport type.

				*N* (%) or Mean (SD)				
Item	Category	Sub-Category	Baseball Group (*N* = 106)	Basketball Group (*N* = 35)	Football/Soccer Group (*N* = 24)	Rugby Group (*N* = 18)	Others Group (*N* = 56)	*p*-Value
Age (years)			17.18 (6.36)	15.57 (6.69)	18.92 (9.69]	19.39 (5.10)	19.34 (12.96)	0.026 *
Sex	Male		101 (95.3)	20 (57.1)	24 (100.0)	17 (94.4)	37 (66.1)	<0.001 *
	Female		5 (4.7)	15 (42.9)	0 (0)	1 (5.6)	19 (33.9)	
Fracture	Zygomatic and zygomatic arch		3 (2.8)	0 (0)	2 (8.3)	2 (11.1)	5 (9.0)	0.155
	Orbital wall (s)		31 (29.2)	3 (8.6)	1 (4.2)	6 (33.3)	11 (19.6)	0.011 *
	Nasal bone		16 (15.1)	5 (14.3)	7 (29.2)	5 (27.8)	5 (9.0)	0.125
	Mandible	Mandibular body	5 (4.7)	0 (0)	0 (0)	1 (5.6)	1 (1.8)	0.453
		Mandibular condyle	0 (0)	0 (0)	0 (0)	0 (0)	2 (3.6)	0.159
		Mandibular body and mandibular condyle	2 (1.9)	0 (0)	0 (0)	0 (0)	0 (0)	0.639
	Panfacial		2 (1.9)	0 (0)	0 (0)	0 (0)	0 (0)	0.639
	Maxilla		8 (7.5)	0 (0)	2 (8.3)	0 (0)	2 (3.6)	0.285
	NOE		6 (5.7)	0 (0)	0 (0)	0 (0)	1 (1.8)	0.252
Non-fracture	Laceration	Mucosa	11 (10.4)	5 (14.3)	2 (8.3)	2 (11.1)	5 (9.0)	0.937
		Skin	1 (0.9)	1 (2.9)	1 (4.2)	0 (0)	3 (5.4)	0.453
	Tooth injury		10 (9.4)	10 (28.6)	5 (20.8)	1 (5.6)	13 (23.2)	0.023 *
	Contusion		5 (4.7)	8 (22.9)	3 (12.5)	0 (0)	5 (9.0)	0.011 *
	Alveolar fracture		6 (5.7)	3 (8.6)	1 (4.2)	1 (5.6)	3 (5.4)	0.960
Referral origin	ECC/ATC		91 (85.8)	24 (68.6)	21 (87.5)	14 (77.8)	37 (66.1)	0.021 *
	Dental/medical clinic		6 (5.7)	5 (14.3)	0 (0)	0 (0)	6 (10.7)	0.117
	Other departments		8 (7.5)	2 (5.7)	1 (4.2)	4 (22.2)	8 (14.3)	0.156
	Direct visit		1 (0.9)	4 (11.4)	2 (8.3)	0 (0)	5 (8.9)	0.040 *
Mode of hospital visit	On foot		96 (90.6)	33 (94.3)	23 (95.8)	16 (88.9)	49 (87.5)	0.726
	Ambulance		10 (9.4)	2 (5.7)	1 (4.2)	1 (5.6)	7 (12.5)	0.680
	Doctor helicopter		0 (0)	0 (0)	0 (0)	1 (5.6)	0 (0)	0.015 *

SD; standard deviation, NOE; naso-orbito-ethmoidal, ECC; emergency and critical care center, ATC; advanced trauma center, * *p* < 0.05.

**Table 4:** Sub-analysis of multivariate logistic regression analysis with baseball-related injury as the dependent variable.

Item	Category	Sub-Category	Multivariate Analysis	
			OR (95% CI)	*p*-Value
Fracture	Orbital wall (s)		4.996 (1.721–14.502)	0.003 *
	Mandible	Mandibular body	9.036 (1.873–43.602)	0.006 *
	Maxilla		4.228 (1.096–16.312)	0.036 *
	NOE		6.993 (1.504–31.953)	0.013 *
Non-fracture	Laceration	Mucosa	0.264 (0.086–0.807)	0.020 *
		Skin	0.096 (0.011–0.872)	0.037 *
	Tooth injury		0.189 (0.061–0.582)	0.004 *

NOE; naso-orbito-ethmoidal, OR; odds ratio, CI; confidence interval, * *p* < 0.05.

## Data Availability

The data supporting the findings of this study are not publicly available because the study protocol did not include a provision for publicly shared data. Approval was obtained from the Medical Research Ethics Committee of Shimane University Faculty of Medicine (kenkyu@med.shimane-u.ac.jp) to request de-identified data.

## Abbreviations

The following abbreviations are used in this manuscript:

ACS	Advanced Trauma Center
CI	Confidence Interval
ED	Emergency Department
NOE	Naso-Orbito-Ethmoidal
OR	Odds Ratio
QoL	Quality of Life
SD	Standard Deviation
TMJ	Temporomandibular Joint
